# Incretin hormones and type 2 diabetes

**DOI:** 10.1007/s00125-023-05956-x

**Published:** 2023-07-11

**Authors:** Michael A. Nauck, Timo D. Müller

**Affiliations:** 1 Diabetes, Endocrinology, Metabolism Section, Medical Department I, Katholisches Klinikum Bochum, St. Josef Hospital, Ruhr-University Bochum, Bochum, Germany; 2 Institute for Clinical Chemistry and Laboratory Medicine, University Medicine Greifswald, Greifswald, Germany; 3 Institute for Diabetes and Obesity, Helmholtz München, Neuherberg, Germany; 4 German Center for Diabetes Research (DZD), München Neuherberg, Germany

**Keywords:** Body weight regulation, Gastric emptying, Gastric inhibitory polypeptide, Glucagon-like peptide-1, Glucagon secretion, Glucose-dependent insulinotropic polypeptide, Incretin, Insulin secretion, Review, Type 2 diabetes

## Abstract

Incretin hormones (glucose-dependent insulinotropic polypeptide [GIP] and glucagon-like peptide-1 [GLP-1]) play a role in the pathophysiology of type 2 diabetes. Along with their derivatives they have shown therapeutic success in type 2 diabetes, with the potential for further improvements in glycaemic, cardiorenal and body weight-related outcomes. In type 2 diabetes, the incretin effect (greater insulin secretory response after oral glucose than with ‘isoglycaemic’ i.v. glucose, i.e. with an identical glycaemic stimulus) is markedly reduced or absent. This appears to be because of a reduced ability of GIP to stimulate insulin secretion, related either to an overall impairment of beta cell function or to specific defects in the GIP signalling pathway. It is likely that a reduced incretin effect impacts on postprandial glycaemic excursions and, thus, may play a role in the deterioration of glycaemic control. In contrast, the insulinotropic potency of GLP-1 appears to be much less impaired, such that exogenous GLP-1 can stimulate insulin secretion, suppress glucagon secretion and reduce plasma glucose concentrations in the fasting and postprandial states. This has led to the development of incretin-based glucose-lowering medications (selective GLP-1 receptor agonists or, more recently, co-agonists, e.g. that stimulate GIP and GLP-1 receptors). Tirzepatide (a GIP/GLP-1 receptor co-agonist), for example, reduces HbA_1c_ and body weight in individuals with type 2 diabetes more effectively than selective GLP-1 receptor agonists (e.g. semaglutide). The mechanisms by which GIP receptor agonism may contribute to better glycaemic control and weight loss after long-term exposure to tirzepatide are a matter of active research and may change the pessimistic view that developed after the disappointing lack of insulinotropic activity in people with type 2 diabetes when exposed to GIP in short-term experiments. Future medications that stimulate incretin hormone and other receptors simultaneously may have the potential to further increase the ability to control plasma glucose concentrations and induce weight loss.

**Graphical Abstract:**

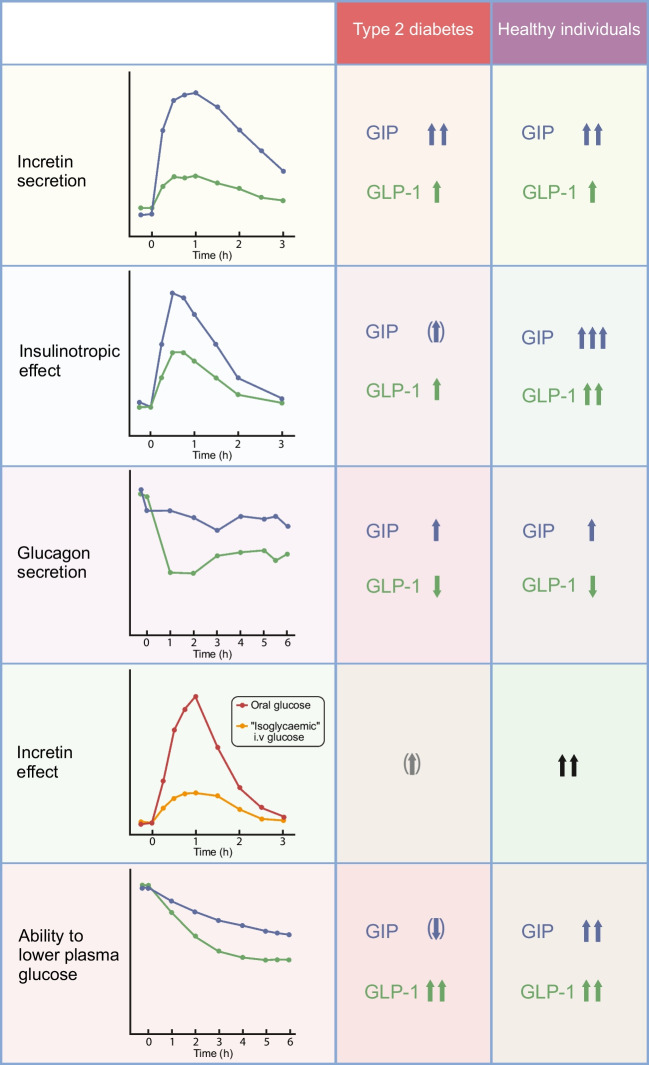

**Supplementary Information:**

The online version of this article (10.1007/s00125-023-05956-x) contains peer-reviewed but unedited supplementary material.

## Incretin hormones in type 2 diabetes: a complex pathophysiological relationship

**Fig. 1:**
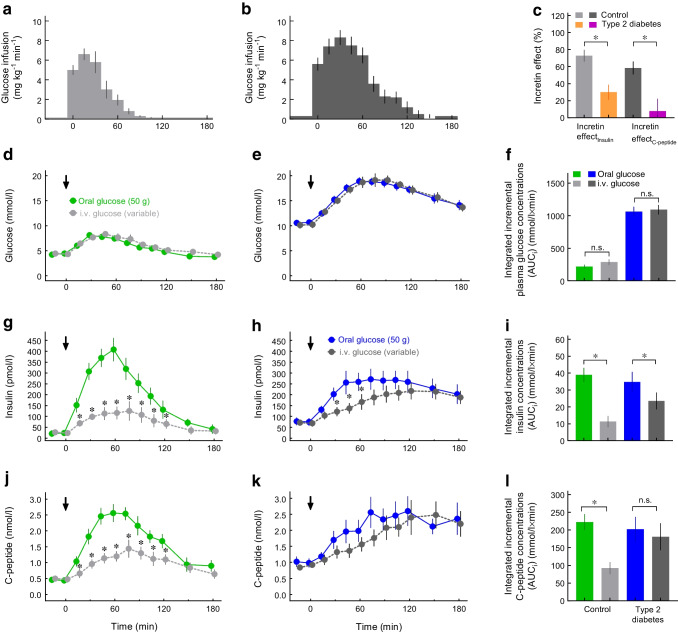
Reduced incretin effect in type 2 diabetes. The incretin effect was quantified in individuals with type 2 diabetes (**b**, **e**, **h**, **l**) and in age- and weight-matched healthy individuals (**a**, **d**, **g**, **k**) by administering oral glucose (50 g) or an i.v. glucose infusion (**a**, **b**), aiming for a matched (‘isoglycaemic’) glycaemic excursion (**d**–**f**) to provide the same degree of hyperglycaemia as the stimulus for insulin secretion. With oral glucose, incretin hormones are released from the gut (not shown) and augment the insulin secretory response (**g**–**i**) and C-peptide levels (**j**–**l**). The difference in insulin secretory response between oral glucose and isoglycaemic i.v. glucose stimulation is the incretin effect, usually expressed as a percentage of the insulin secretory response after oral glucose (**c**). This measure of the incretin effect is greatly reduced in patients with type 2 diabetes, whether calculated from insulin or C-peptide responses (**c**). *p*<0.05; n.s., not significant. Data from [2, 15]. (**a**, **b**, **d**, **e**, **g**, **h**, **k**, **l**) adapted from [2] with permission from Springer Nature

The extraordinary success of incretin-based glucose-lowering medications in type 2 diabetes,, which are mainly based on properties of the incretin hormone glucagon-like peptide-1 (GLP-1) [1], has reinforced interest in the physiology and pathophysiology of incretin hormones more generally. The main finding when studying the secretion and action of incretin hormones (glucose-dependent insulinotropic polypeptide [GIP] and GLP-1) in individuals with type 2 diabetes is a reduced incretin effect. The term ‘incretin effect’ refers to the greater stimulation of insulin secretion with oral glucose than i.v. glucose, even when the same amount (e.g. 75 g) is administered, or, more appropriately, when the glycaemic excursions are similar (‘isoglycaemic’ i.v. glucose infusion), which is the typical finding in metabolically healthy individuals (Fig. 1a,d,j,k). In contrast, in people with type 2 diabetes, insulin secretory responses show the typical slow rise to a peak, occurring later than in healthy individuals, after oral glucose ingestion, but this response is only marginally higher than that to ‘isoglycaemic’ i.v. glucose (Fig. 1b,e,h,l), indicating a substantially reduced or, in some individuals, absent incretin effect [2–4].

In this review, we summarise current knowledge regarding the secretion and insulinotropic action of GIP and GLP-1, their contribution to the incretin effect, and their effects on glucagon secretion, gastric emptying, appetite and energy intake in people with type 2 diabetes and healthy individuals. In addition, we discuss the therapeutic potential of incretin hormones as parent compounds for glucose-lowering medications, based on short-term proof-of-principle studies showing the potential of exogenously administered GIP and GLP-1 (or their combination) to stimulate insulin secretion, reduce glucagon secretion and decelerate gastric emptying. However, it should be noted that long-term stimulation of the same GIP and GLP-1 receptors may elicit effects that differ in quantitative or even qualitative terms.

## Secretion of incretin hormones in type 2 diabetes

**Fig. 2:**
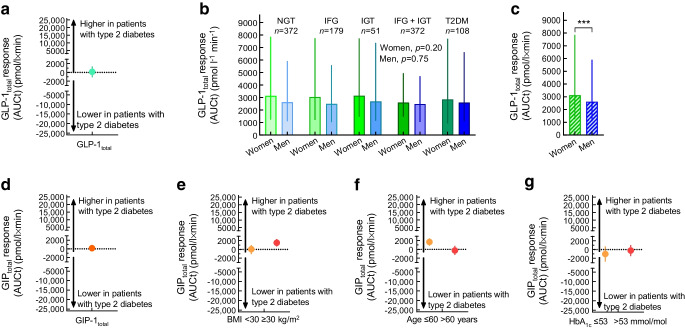
Secretion of GLP-1 (**a**–**c**) and GIP (**d**–**g**) in individuals with type 2 diabetes relative to healthy individuals. (**a**) Absence of a significant difference in GLP-1_total_ plasma responses following nutrient intake (oral glucose, liquid or solid meals) between individuals with type 2 diabetes and healthy individuals (meta-analysis by Calanna et al [8]). (**b**) Lack of significant differences in GLP-1_total_ plasma responses following an oral glucose load across categories of glucose tolerance (including screen-detected type 2 diabetes) in women and men [13]. Differences between women and men were not significant for any of the subcategories based on glucose tolerance status. (**c**) Higher GLP-1_total_ plasma responses following an oral glucose load in women (*n*=683) than in men (*n*=779) across all categories of glucose tolerance combined [13]. (**d**) Absence of a significant difference in GIP_total_ plasma responses following nutrient intake (oral glucose, liquid or solid meals) between individuals with type 2 diabetes and healthy individuals (meta-analysis by Calanna et al [9]). (**e**–**g**) Subgroup analyses (meta-regression analysis) indicating (non-significant trends for) relatively higher GIP_total_ plasma responses following nutrient intake (oral glucose, liquid or solid meals) in individuals with type 2 diabetes vs healthy individuals in the presence of a higher BMI (≥30 kg/m^2^; **e**), lower age (≤60 years; **f**) and lower HbA_1c_ level (≤53 mmol/mol [≤7.0%]); **g**) [9]. Incretin hormone secretion was systematically assessed measuring total GIP and GLP-1 concentrations (intact GIP and GLP-1 plus metabolites generated by proteolytic degradation). ***p<0.001. AUC_t_, AUC above a concentration of 0; GLP-1_total_/GIP_total_, GLP-1/GIP levels including breakdown products recognised in the assay; T2DM, type 2 diabetes

One possible explanation for the reduced incretin effect in type 2 diabetes is a lack or shortage of incretin hormone secretion. Incretin hormone secretion is assessed following nutrient intake by measuring plasma concentration profiles of GIP_total_ and GLP-1_total_, which are the sums of intact GIP (1–42) or GLP-1 (7–36 amide [amidated form] or 7–37 [glycine-extended form]) and their metabolites generated by dipeptidyl peptidase-4 (DPP-4)-mediated degradation (GIP [3–42] and GLP-1 [9–36 amide and 9–37]). However, when using novel, highly specific assays, the secretion of both GIP and GLP-1 does not appear to be systematically different in those with type 2 diabetes (Fig. 2). Some earlier studies suggested the presence of hypersecretion of GIP [5] and hyposecretion of GLP-1 [6, 7] in type 2 diabetes, but meta-analyses show no apparent systematic differences in secretion between people with type 2 diabetes and healthy individuals [8, 9]. The characteristics of individuals with and without type 2 diabetes from these meta-analyses [8, 9] are summarised in ESM Table 1.

The heterogeneous findings reported in the individual studies contributing to these meta-analyses [9] are probably explained by the large inter-individual variation in the secretion of GIP and GLP-1 [10]. Interestingly, people with low levels of GIP may also have low levels of GLP-1 and vice versa [10].

The early cross-sectional study of GLP-1 secretion by Toft-Nielsen et al [7], which reported progressively diminishing GLP-1 responses after mixed meal stimulation in those with impaired glucose tolerance (IGT) and type 2 diabetes, has often been interpreted as indicating longitudinal changes, from normal GLP-1 secretion in healthy individuals to somewhat reduced GLP-1 secretion in those with IGT to substantially reduced GLP-1 secretion in people with type 2 diabetes. Based on these data, it was predicted that, in long-standing type 2 diabetes, GLP-1 secretion subsides altogether. This study [7] was published before the initial clinical trials of GLP-1 receptor agonists [11, 12] and allowed this novel therapy to be viewed as a replacement for what appeared to be lacking in people with type 2 diabetes.

In a large cross-sectional study of 1082 participants with a well-characterised glucose tolerance status, total GLP-1 responses (above a concentration of zero) induced by oral glucose ingestion were similar for all categories of glucose tolerance, from normal to type 2 diabetes, in both women and men [13]. However, for each glucose tolerance category, the response was numerically, but not significantly, higher in women (Fig. 2b). When all participants was analysed together, that is, independently of their glucose tolerance status, there was a significant difference of 18% between men and women (Fig. 2c). It should be noted that type 2 diabetes was detected by screening so this study does not provide information on later-stage, advanced type 2 diabetes [13]. A secondary analysis of integrated incremental GLP-1 responses (above baseline concentrations) showed a slightly different result. In women, but not in men, there was a significant difference between different categories of glucose tolerance (lower response in those with impaired fasting glucose [IFG]/IGT vs normal glucose tolerance (NGT), isolated IFG or IGT, and screen-detected type 2 diabetes; ESM Fig. 1), but no significant difference between those with type 2 diabetes and those with NGT. Total AUCs for GLP-1 after oral glucose increased significantly with increasing age and decreased significantly with increasing BMI and waist circumference, with higher values in women throughout the observed ranges of age, BMI and waist circumference [13]. Regarding BMI, this relationship has also been described by Muscelli et al [14].

With regard to GIP, although overall secretion as assessed from a meta-analysis of nutrient-stimulated secretion (oral glucose or mixed meals) appears to be similar for those with NGT and those with type 2 diabetes (Fig. 2d), there were non-significantly higher plasma GIP responses in type 2 diabetes for those with a higher BMI (Fig. 2e), who were younger (Fig. 2f) and with higher HbA_1c_ levels (Fig. 2g) [9].

## Insulinotropic action of GIP and GLP-1 in type 2 diabetes

The mechanisms of action of GIP and GLP-1 are essentially related to the distribution of the GIP and GLP-1 receptors in tissues and cells. Several recent reviews have provided details on GIP and GLP-1 receptor distribution and the physiology of incretin hormones [15, 16].

**Fig. 3:**
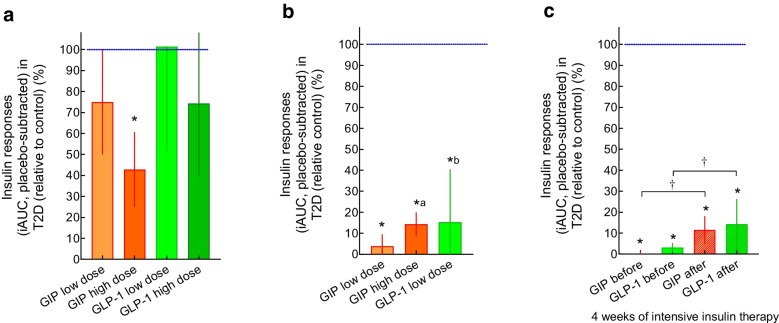
Comparison of insulinotropic effects (integrated incremental insulin responses [iAUC]) of GIP (orange) and GLP-1 (green) in participants with type 2 diabetes and healthy control participants, whose mean values were taken as 100% (blue line). For details of the experimental conditions and characteristics of participants, see ESM Table 2. (**a**) Exogenous GIP and GLP-1 at 0.8 and 2.4 vs 0.4 and 1.2 pmol kg^−1^ min^−1^, respectively, for 1 h each during hyperglycaemic clamp experiments aiming at 8.0 mmol/l plasma glucose levels [22]. (**b**) Exogenous GIP and GLP-1 at 4 and 16 pmol kg^−1^ min^−1^ (only in individuals with type 2 diabetes) vs 1.0 pmol kg^−1^ min^−1^, respectively, for 2 h each during hyperglycaemic clamp experiments aiming at 15.0 mmol/l plasma glucose levels [23]. (**c**) Exogenous GIP and GLP-1 at 1.5 vs 0.5 pmol kg^−1^ min^−1^, respectively, for 2 h each during hyperglycaemic clamp experiments aiming at 15.0 mmol/l plasma glucose levels, both before and after a 4 week course of intensified insulin therapy to provide near-normoglycaemic plasma glucose concentrations [24]. ^a^Results with a GIP infusion rate of 16 pmol kg^−1^ min^−1^ in participants with type 2 diabetes are compared with those obtained with 4 pmol kg^−1^ min^−1^ in control participants, as this high dose was not studied in control participants. ^b^GLP-1 was studied in only one healthy control participant, whose value was taken as 100%. *p<0.05 vs healthy control participants. †*p*<0.05 comparing results before and after intensive insulin treatment in participants with type 2 diabetes. T2D, type 2 diabetes

After the discovery of GIP in the early 1970s, its insulinotropic actions were initially studied in healthy rodents [17, 18] and healthy humans [19]. At this time, GIP was isolated and purified from animal sources (usually porcine gut mucosa) and was not widely available. Therefore, it was not until the mid-1980s that the results of administering exogenous (porcine) GIP to individuals with type 2 diabetes were reported [20]. Insulin and C-peptide levels were measured at plasma glucose concentrations of ~11 mmol/l in a small number of individuals with type 2 diabetes and at plasma glucose concentrations of ~5 mmol/l in healthy individuals. When administering exogenous porcine GIP, C-peptide levels rose slightly in those with type 2 diabetes, but plasma glucose levels did not change [20]. In 1987, Krarup et al reported a comparison of GIP effects in healthy individuals and individuals with type 2 diabetes clamped at the same degree of hyperglycaemia. While a substantial insulinotropic effect (necessitating a sharp rise in glucose infusion rates to maintain the clamp target glucose concentrations) was observed in healthy individuals, a very minor response was seen in those with type 2 diabetes, in particular with respect to the effects on glucose metabolism (infusion rates) [21]. Those with type 1 diabetes also did not display an insulinotropic response to exogenous porcine GIP [21]. The finding of a greatly reduced insulinotropic response to (physiological replacement and supra-physiological doses of) exogenous GIP was later confirmed once human (synthetic) GIP became available [22]. Vilsbøll et al subsequently pointed out that the late phase of the insulinotropic response is particularly impaired in type 2 diabetes [23]. Several studies that have analysed the insulinotropic response to exogenous GIP in individuals with type 2 diabetes compared with healthy individuals under hyperglycaemic clamp conditions are summarised in Fig. 3. Participant characteristics and study conditions are provided in ESM Table 2. Insulin secretory responses (insulin and C-peptide rises above baseline) during the administration of exogenous human synthetic GIP were uniformly much smaller in those with type 2 diabetes than in healthy individuals [22–24].

The insulinotropic effects of the molecular form(s) of GLP-1 produced in intestinal L cells on the rodent pancreas were first published in 1987 (GLP-1 [7–36 amide], amidated GLP-1 [25]; GLP-1 [7–37], glycine-extended GLP-1 [26]), followed shortly after by the insulinotropic effects in healthy humans [27]. A study comparing the effects of GIP and GLP-1 in perfused pancreases from healthy rats and a streptozotocin-induced rat model of diabetes suggested a similar impairment regarding the insulinotropic actions of GLP-1 as previously shown for GIP in a model of human type 2 diabetes [28]. The fact that results from animal models of type 2 diabetes did not predict results obtained in human type 2 diabetes patients points to the fact that these models resemble human type 2 diabetes in an imperfect manner because of differences in essential pathophysiological prerequisites.

The effects of exogenous GLP-1 (7–36) amide in individuals with type 2 diabetes were first published in 1993 (and compared with those of GIP; see above) [22]. In contrast to the results from animal models and the effects of exogenous GIP, GLP-1, both at a physiological dose (leading to plasma concentrations similar to those observed with nutrient stimulation) and at a threefold higher, more ‘pharmacological’ dose, augmented the insulin secretory response in individuals with type 2 diabetes (Fig. 3a; [22]). This response (integrated incremental C-peptide, representing the insulin secretory response) was ~70% of the response in healthy individuals, while the comparable figure for GIP was ~40%. The preserved insulinotropic response to exogenous GLP-1 in type 2 diabetes has been confirmed in subsequent studies [23, 29–32].

Compared with healthy individuals, incretin-induced insulin secretory responses in type 2 diabetes seem to be reduced more (Fig. 3), with less well-controlled plasma glucose concentrations (indicated by lower baseline HbA_1c_ levels, fewer diabetes medications; ESM Table 2). The study by Nauck et al [22] was performed using 8 mmol/l hyperglycaemic clamps, whereas the other studies typically used 15 mmol/l clamps [23, 24]. As hyperglycaemia and incretin actions in combination stimulate insulin secretion, the latter studies probably inform about differences in glucose-induced insulin secretion rather than differences in incretin-stimulated insulin secretion [23, 24], which is the major determinant of insulin secretion at more physiological glucose concentrations [22]. Participant characteristics and experimental conditions for the studies shown in Fig. 3 are provided in ESM Table 2.

A detailed study on how different infusion rates of GLP-1 affect the slope relating (clamped) plasma glucose concentrations to GLP-1-induced insulinotropic responses showed a linear dose–response relationship for both healthy individuals and those with type 2 diabetes, but the slope was three to five times less steep in type 2 diabetes [33]. This phenomenon has sometimes been referred to as ‘GLP-1 resistance’, although this dose–response relationship does not preclude clinically significant reductions in plasma glucose with exogenous GLP-1 (see above).

## GIP and GLP-1 effects on glucagon secretion in type 2 diabetes

**Fig. 4:**
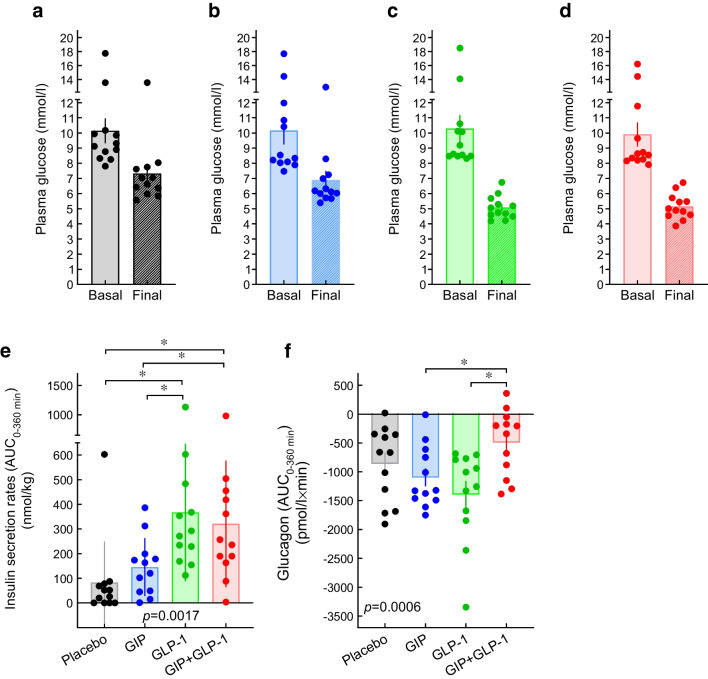
Plasma glucose at baseline and 6 h after the infusion of exogenous placebo (**a**), GIP (**b**), GLP-1 (**c**) and GIP + GLP-1 (**d**) in individuals with type 2 diabetes in whom fasting hyperglycaemia was provoked by the omission of the long-acting insulin injection the night before each study (crossover design). Insulin (**e**) and glucagon (**f**) responses are also shown as AUCs above (insulin) and below (glucagon) baseline concentrations. Data are redrawn from Mentis et al [41]. Plasma glucose at baseline was not significantly different between the different study days (*p*=0.59), while plasma glucose concentrations at the end of the experiments differed significantly: placebo vs GIP, *p*=0.71; placebo vs GLP-1, *p*<0.001; placebo vs GIP + GLP-1, *p*<0.001; GIP vs GLP-1, *p*<0.001; GIP vs GIP + GLP-1, *p*<0.001; GLP-1 vs GIP + GLP-1, *p*>0.99. For the other comparisons, *p<0.05. Overall *p* values are presented for ANOVA comparing all experimental conditions

GIP stimulates glucagon secretion in healthy individuals and those with type 2 diabetes, especially at lower plasma glucose concentrations [34–37], whereas the insulinotropic actions are more prominent during hyperglycaemia [35, 36]. Exogenous GIP increased plasma glucagon responses to a mixed meal in individuals with type 2 diabetes and apparently led to a transient worsening of plasma glucose excursions [37], despite some (limited) evidence of insulinotropic activity during the early phase after meal ingestion. GLP-1 suppresses glucagon secretion in healthy individuals and those with type 2 diabetes, especially at higher plasma glucose concentrations [22, 29, 38] (while the counter-regulatory glucagon response in the case of hypoglycaemia remains unaffected [39]). The suppression of glucagon secretion by GLP-1 contributes to its glucose-lowering effects [40]. Interestingly, the suppression of glucagon by exogenous GLP-1 in type 2 diabetes was antagonised by concomitant administration of GIP, which alone did not significantly affect plasma glucagon concentrations (Fig. 4f) [41].

Taken together, these studies show that changes in glucagon concentration in response to GIP and GLP-1 depend on plasma glucose and insulin concentrations. Given the obvious differences in these confounders between healthy individuals and those with type 2 diabetes, there does not seem to be a fundamental difference in how GIP and GLP-1 influence glucagon secretion in healthy individuals compared with those with type 2 diabetes. Accordingly, the glucagonostatic effect of GLP-1 is fully preserved in those with type 2 diabetes [22, 42], and GIP also stimulates glucagon secretion in those with type 2 diabetes [43].

## GLP-1 and gastric emptying in type 2 diabetes

GLP-1 decelerates gastric emptying in those with type 2 diabetes as well as in healthy individuals [44]. Even low doses (leading to close to physiological plasma concentrations) have this effect and contribute to reduced post-meal glycaemic excursions [45]. GIP does not affect gastric emptying in healthy individuals or those with type 2 diabetes [46].

The effect on gastric emptying is the only effect of GLP-1 (receptor agonists) where tachyphylaxis develops after a relatively short duration of treatment [47]. This is illustrated by the differential effects of short- vs long-acting GLP-1 receptor agonists on gastric emptying after 8 weeks’ treatment [48]. Other effects (relevant for glycaemic control and body weight reduction) are not subject to tachyphylaxis or desensitisation.

## GIP and GLP-1 effects on fasting and postprandial lipids/lipoproteins in type 2 diabetes

While the effects of exogenous GIP at physiological or pharmacological doses on fasting and postprandial lipids/lipoproteins in type 2 diabetes have not been explicitly published, Stensen et al did not find any changes in basal or postprandial concentrations of NEFAs and glycerol, triacylglycerol, and HDL-, VLDL- and LDL-cholesterol when administering a GIP receptor antagonist (GIP [3–30] NH_2_) in individuals with type 2 diabetes during a (liquid) meal test, thus ruling out acute effects of endogenously secreted GIP [49]. Effects of longer term exposure to exogenous GIP or GIP receptor antagonists on lipid parameters have not been reported, as there are no available GIP receptor agonists or antagonists with suitable pharmacokinetic properties for prolonged exposure in human studies.

**Fig. 5:**
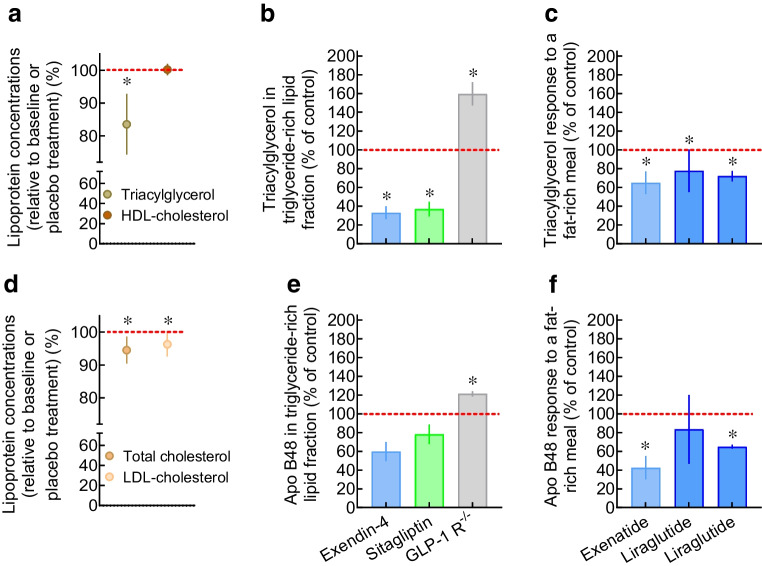
Incretin hormone GLP-1 and its derivatives (GLP-1 receptor agonists) and fasting and postprandial lipids/lipoproteins. (**a**, **d**) Effects of GLP-1 receptor agonists on fasting triacylglycerol and HDL-cholesterol concentrations (**a**) and total and LDL-cholesterol concentrations (**d**) in individuals with type 2 diabetes (meta-analysis by Song et al [50]). (**b**, **e**) Effects of exendin-4 (a GLP-1 receptor agonist), sitagliptin (a DPP-4 inhibitor) and a transgenic GLP-1 receptor knockout (GLP-1 R^–/–^) on postprandial triacylglycerol (**b**) and apo B48 (**e**) levels (relative to untreated control animals) in non-diabetic mice ([52]). (**c**, **f)** Effects of GLP-1 receptor agonists (exenatide, left-hand bars [56]; liraglutide, right-hand bars [57], centre bars [58]) on triacylglycerol (**c**) and apo B48 (**f**) levels in individuals with type 2 diabetes following a fat-rich meal (relative to placebo treatment). The data displayed in this figure indicate the effects of GLP-1 receptor agonism on fasting lipoprotein particles and, in particular, on the postprandial formation of triacylglycerol-rich lipoproteins (chylomicrons), which is reduced significantly and substantially. *p<0.05 relative to untreated (placebo-treated) control participants

Meta-analyses of clinical trials of GLP-1 receptor agonists showed a reduction in fasting triacylglycerol and total cholesterol and LDL-cholesterol concentrations in type 2 diabetes, while concentrations of HDL-cholesterol were unchanged (Fig. 5a,d) [50]. These changes may at least partially be due to weight loss induced by such treatment. GLP-1 also reduced NEFAs in fasting hyperglycaemic individuals with type 2 diabetes [29, 41], mainly during the period when insulin concentrations were elevated owing to the insulinotropic action of GLP-1. This effect, therefore, is indirect and mediated by insulin inhibiting lipolysis in adipose tissue.

GLP-1 is physiologically involved in limiting the formation and secretion into the lymph of chylomicrons formed during the process of triacylglycerol absorption from the gut, as a GLP-1 receptor antagonist, exendin (9–39), increased triacylglycerol and apolipoprotein B-48 (apoB48) concentrations after an intestinal lipid load in rats [51], and the GLP-1 receptor agonist exendin-4 reduced triacylglycerol and apoB48 concentrations [52] (Fig. 5b,e). This indicates that physiological concentrations of GLP-1 exert a tonic inhibition of chylomicron formation. This is also indicated by the fact that DPP-4 inhibitors reduce postprandial triacylglycerol and apoB48 responses, although they only double the concentrations of intact, biologically active GLP-1 [53, 54]. All GLP-1 receptor agonists studied for effects on postprandial triacylglycerol-rich lipoproteins and apoB48 production have provided evidence of a reduction compatible with the effects on chylomicron formation described above (see [55]). Figure 5c,f shows the effects of exenatide and liraglutide on triacylglycerol and/or apoB48 concentrations, respectively, in people with type 2 diabetes [56–58]. Lixisenatide also increased chylomicron triacylglycerol clearance [58], whereas dulaglutide did not reduce postprandial apoB48 concentrations after a meal in a small study in Japanese people with type 2 diabetes [59].

Thus, while longer term treatment with GLP-1 receptor agonists clearly reduces fasting triacylglycerol and LDL-cholesterol levels (perhaps, in part, related to body weight reduction) and suppresses the formation of chylomicrons and postprandial increases in triacylglycerol, lipoprotein concentrations do not seem to be affected by antagonising physiological concentrations of endogenously secreted GIP in type 2 diabetes.

## Differential effect on elevated plasma glucose concentrations of exogenous GIP and GLP-1 in type 2 diabetes

The question arises whether the ability of GIP and/or GLP-1 to augment glucose-induced insulin secretion (Fig. 3) translates into an ability to lower plasma glucose in individuals with type 2 diabetes. In type 2 diabetes, GIP has a limited ability to stimulate insulin secretion during hyperglycaemia [21, 22] and does not significantly reduce plasma glucose concentrations [20, 41]. In contrast, 1.0–1.2 pmol kg^−1 ^min^−1^ of exogenous GLP-1 (7–36) amide normalised plasma glucose concentrations within 4–5 h in individuals with type 2 diabetes, starting with fasting hyperglycaemia during continued fasting [29, 31, 32, 41]. GLP-1 (7–36 amide) and GLP-1 (7–37) were equally effective [32], and those with type 2 diabetes of long duration and treated with insulin after well-documented sulfonylurea secondary failure responded (despite a reasonable assumption that they would have a relatively greater reduction in beta cell mass and function) [31]. In such short-term experiments, even very high (‘pharmacological’) doses of GIP had no beneficial effects indicating a therapeutic potential for GIP, while exogenous GLP-1 uniformly lowered plasma glucose. Figure 4 shows the results of a study investigating the effects of GIP, GLP-1 and GIP/GLP-1 in combination on plasma glucose concentrations in patients with type 2 diabetes [41]. In summary, only GLP-1 (alone or in combination with GIP) stimulated insulin secretion (insulin, C-peptide and insulin secretion rates were calculated by deconvolution) and lowered plasma glucose in previously hyperglycaemic individuals with type 2 diabetes; GIP alone had no significant effects.

## Potential explanations for the differential reduction in GIP and GLP-1 insulinotropic activity in type 2 diabetes

As the mechanisms for the stimulation of insulin secretion by GIP and GLP-1 are very similar and mainly involve the generation of cAMP through effects on G proteins coupled to the binding of ligands to GIP and GLP-1 receptors [60], a marked reduction in insulinotropic activity by GIP but widely preserved activity by GLP-1 in type 2 diabetes is surprising and not easily explained. Some animal studies have found that the expression of GIP (and, to a lesser degree, GLP-1) receptors in pancreatic beta cells is (reversibly) reduced during hyperglycaemia [61, 62]. In line with these studies, the insulinotropic effectiveness of exogenous GIP (and GLP-1) was improved in individuals with type 2 diabetes after 4 weeks’ treatment with an intensive insulin regimen, leading to a near-normalisation of plasma glucose concentrations [24, 63], also indicating some association of hyperglycaemia with reduced action of incretin hormones, in particular GIP. These findings are illustrated in Fig. 3c. However, this improvement in insulinotropic activity of GIP and GLP-1, although significant, did not lead to the same degree of insulinotropic activity as in healthy individuals and, thus, represents a partial improvement, not full normalisation. This finding also challenges the presumed association of hyperglycaemia with reduced GIP and GLP-1 receptor expression in pancreatic beta cells.

Another potential explanation for the differential reduction in insulinotropic potency of GIP (much impaired) and GLP-1 (mildly reduced) in type 2 diabetes is that GLP-1 can activate the G proteins G_aq_ and G_as_, whereas GIP can only activate G_as_. In individuals with type 2 diabetes with chronic hyperglycaemia or in those treated with sulfonylureas, pancreatic beta cells are chronically depolarised, which in turn leads to a switch from G_as_ to G_aq_ as the major pathway for stimulating insulin secretion [64]. Hence, in mice, GIP receptor signalling is progressively impaired under such conditions, while GLP-1 receptor signalling remains (partially) active through G_aq_ [64]. It is not known, however, whether these findings are specific to the transgenic mouse models used in this study or represent a more universal phenomenon. Thus, their relevance to the reduced incretin effect in human type 2 diabetes can be questioned. As several animal studies using streptozotocin-induced rat and *ob/ob* (leptin-deficient) mouse models of diabetes have shown insulinotropic actions of GIP and/or GIP receptor agonists [65–67], these models of type 2 diabetes may not be suitable to study the phenomenon of reduced insulinotropic activity of GIP in human type 2 diabetes.

## Determinants of the quantitative impact of the incretin effect in type 2 diabetes

Generally speaking, the incretin effect is reduced or absent in type 2 diabetes (Fig. 1) because of the impaired insulinotropic action of endogenously secreted GIP, as can be inferred from the reduced insulinotropic activity of exogenously administered GIP (Fig. 2) [22–24, 63]. The partially preserved insulinotropic activity of GLP-1 in type 2 diabetes is probably not sufficient to support a sizeable incretin effect, as GLP-1 responses after oral glucose are smaller than those for GIP [4]. Even in healthy individuals (with a prominent incretin effect), GLP-1 contributes only a minor proportion of the incretin effect, as shown by using specific GIP and GLP-1 receptor antagonists [68–70].

**Fig. 6:**
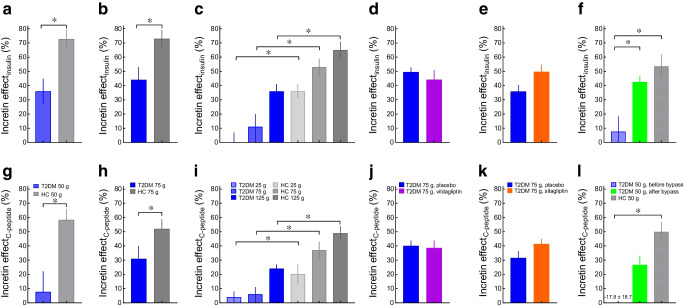
Quantification of the incretin effect in participants with type 2 diabetes (T2DM) and healthy control participants (HC) based on the measurement of insulin (**a**–**f**) and C-peptide (**g**–**l**). Data from (**a**, **g**) Nauck et al [2] (oral glucose load 50 g); (**b**, **h**) Knop et al [71] (oral glucose load 75 g); (**c**, **i**) Bagger et al [3] (oral glucose loads of 25, 75 and 125 g); (**d**, **j**) Vardarli et al [72] (oral glucose load 75 g; after placebo and vildagliptin treatment); (**e**, **k**) Vardarli et al 2014 [73] (oral glucose load 75 g; after placebo and sitagliptin treatment); and (**f**, **l**) Laferrere et al [74] (before and after Roux-en-Y gastric bypass in individuals with obesity and type 2 diabetes). A negative value for the incretin effect before Roux-en-Y gastric bypass determined by measuring C-peptide levels is indicated by presenting the results as numbers (mean ± SEM) in panel **l**. (**a**–**c**, **g**–**i**) adapted from [4] with permission from Elsevier. *p<0.05

The results of several studies demonstrating a significantly reduced incretin effect in type 2 diabetes are shown in Fig. 6 [2, 3, 71–74]. Participant characteristics and experimental details for the studies shown in Fig. 6 are provided in ESM Table 3. As in healthy individuals, there is a dose-dependency regarding the amount of glucose ingested, with greater incretin effects at higher doses, but at all glucose loads the incretin effect is significantly reduced in those with type 2 diabetes compared with healthy individuals (Fig. 6c,i) [3]. In a similar way, the secretion of GIP and GLP-1 is greater at higher doses, which does not affect peak GIP and GLP-1 concentrations, but leads to a prolonged elevation in GIP and GLP-1 plasma concentrations after oral glucose administration both in healthy individuals and in those with type 2 diabetes. Accordingly, the period with greater insulin secretion with oral vs ‘isoglycaemic’ i.v. glucose is prolonged at higher glucose doses, leading to a greater incretin effect with 125 vs 75 vs 25 g glucose loads [3].

Furthermore, it has also been shown that the incretin effect is decreased in people with obesity and NGT relative to lean individuals with NGT [75]. In line with this, the incretin effect is inversely correlated with glucose tolerance and BMI in a mutually independent and additive manner [14].

Inhibitors of DPP-4 lower glucose levels in individuals with type 2 diabetes because they prevent the degradation and inactivation of the intact biologically active molecular forms of GIP and GLP-1 [76]. However, studies quantifying the incretin effect in type 2 diabetes before and after treatment with either vildagliptin [72] or sitagliptin [73] (DPP-4 inhibitors) surprisingly do not find an augmented effect (Fig. 6d,e,k,l), mainly because GLP-1 concentrations and insulin secretion are elevated both with oral glucose and with ‘isoglycaemic’ glucose infusions, even though, in the latter case, these GLP-1 concentrations remain in the low (basal) range [72, 73].

The only condition that has been associated with a significant increase in the incretin effect in individuals with type 2 diabetes is bariatric surgery (Roux-en-Y gastric bypass; Fig. 6f,m) [74]. This is the result of an increase in GLP-1 secretion [74], as nutrients are delivered rapidly from a small gastric remnant (without a pylorus) to lower sections of the jejunum, where L cells producing GLP-1 (and peptide YY [PYY]) are more prominent [77]. GIP responses are also elevated (unexpectedly, as K cells in the duodenum are less exposed to nutrients after gastric bypass) [74]. Increases in PYY levels may also explain the healthier microstructure and function of the endocrine pancreas [78].

Two studies have examined the role of race and ethnicity in the incretin effect. A study from South Korea suggested that, in an Asian population with type 2 diabetes, the incretin effect may not be reduced [79], in contrast to the reduction seen in white populations with type 2 diabetes. However, this was not confirmed in a subsequent study performed in Malaysia [80].

Finally, the incretin effect is quantified by comparing insulin secretory responses to oral glucose (a ‘strong’ stimulus for insulin secretion) and ‘isoglycaemic’ i.v. glucose (a ‘weaker’ stimulus for insulin secretion). It has been argued that individuals with type 2 diabetes, who have well-characterised reductions in beta cell mass and function [81], may already secrete insulin maximally with the weaker stimulus so that the secretory response cannot be augmented further with a stronger stimulus.

The following section discusses novel therapeutic concepts based on what is known about the (patho-)physiology of incretin hormones in type 2 diabetes.

## Therapeutic potential for combined GIP and GLP-1 receptor agonism?

Over the last few years we have witnessed remarkable progress in the development of long-acting GLP-1 receptor agonists, including the generation of unimolecular peptides that simultaneously activate the receptors for GLP-1 and GIP.

**Fig. 7:**
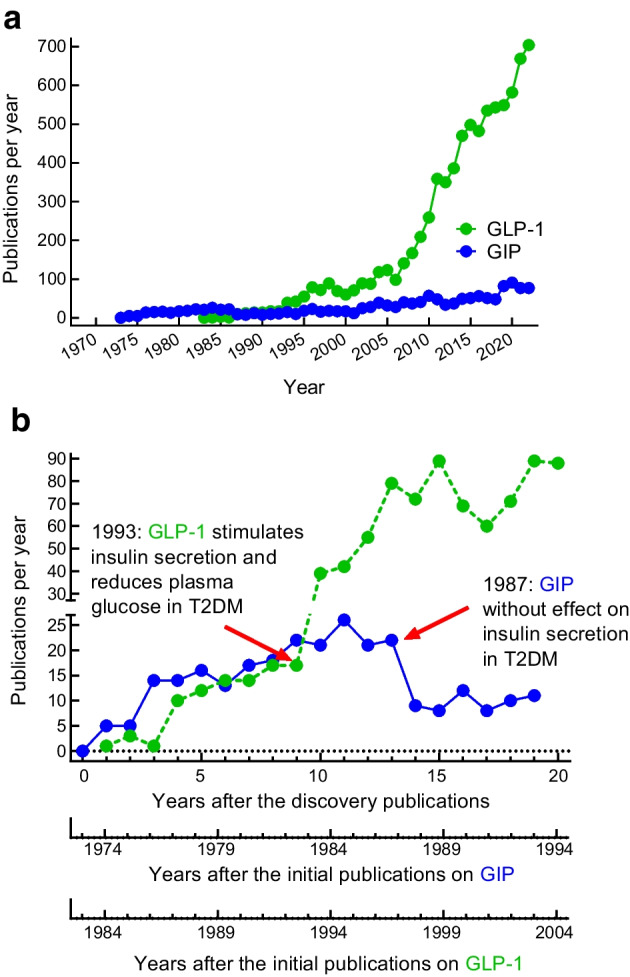
Numbers of publications per year with glucose-dependent insulinotropic polypeptide/gastric inhibitory peptide/GIP and glucagon-like peptide-1/GLP-1 in their title. (**a**) Publications per calendar year (retrieved from PubMed). (**b**) Publications per year following the initial discoveries of GIP and GLP-1. The numbers of publications were comparable for the first 9–13 years after the first descriptions, but changed substantially after the characterisation of insulinotropic activity in individuals with type 2 diabetes (decrease after the initial publication showing reduced effectiveness of GIP [21]; increase after the initial publications showing preserved activity of GLP-1 [22, 29]). T2DM, type 2 diabetes

Initial short-term studies that found that exogenous GIP barely stimulates insulin secretion in people with type 2 diabetes (Fig. 3) [21–23] and does not induce a substantial reduction in plasma glucose concentrations in hyperglycaemic individuals with type 2 diabetes (Fig. 4) [41, 82] did not support the idea that GIP has therapeutic potential for the treatment of type 2 diabetes. After the publication of the first report that porcine GIP has little insulinotropic activity in people with type 2 diabetes [21], the interest in GIP dramatically decreased, as shown by the number of publications on GIP after 1987 (Fig. 7). In contrast, reports of the ability of GLP-1 to stimulate insulin secretion at elevated plasma glucose concentrations [22, 23] and its potential to lower plasma glucose concentrations in hyperglycaemic individuals with type 2 diabetes (Fig. 4) [22, 30–32, 41] led to an increase in the number of publications on GLP-1 (Fig. 7) and to the successful development of GLP-1 receptor agonists [1]. Recently, renewed discussion on the therapeutic potential of GIP in the long-term treatment of type 2 diabetes has been triggered by clinical findings with the dual GIP/GLP-1 receptor agonist tirzepatide [83].

## Future developments and open research questions regarding incretin hormones and their therapeutic analogues in type 2 diabetes

Both glycaemic control (often leading to normal HbA_1c_ levels) and body weight reduction are significantly better with tirzepatide than with the most efficacious selective GLP-1 receptor agonists (e.g. semaglutide) [83, 84]. Engagement of GIP in a biochemical liaison with GLP-1 seems at first glance counterintuitive, given that the insulinotropic effects and the incretin effect of GIP are severely reduced in people with type 2 diabetes [2, 22] (see earlier), and that GIP receptor-deficient mice are lean and protected from diet-induced obesity [85]. GIP enhances adipocyte triacylglycerol deposition by increasing adipocyte blood flow and accelerating lipoprotein lipase-induced lipid storage [15, 86, 87], and both should correlate with weight gain. However, near-normalisation of hyperglycaemia using insulin (which is potentially also possible through GLP-1-mediated improvements in glycaemic control) may at least partially restore the insulinotropic action of GIP in people with type 2 diabetes (Fig. 3 [24]), and co-administration of GIP with a GLP-1 receptor agonist leads to greater weight loss in diet-induced obese (DIO) mice relative to treatment with the GLP-1 receptor agonist alone [88].

To what extent, if any, GIP contributes to tirzepatide-induced weight loss is the subject of intense scientific investigation. Preclinical studies (animal experiments using shrews, a species that has the ability to vomit) show that long-acting GIP receptor agonists attenuate GLP-1-induced nausea and emesis [89] while also acting on central nervous system GIP receptors to decrease body weight through inhibition of food intake [90]. Consistent with this, even ligand-independent activation of GIP receptor-expressing neurons decreases food intake in mice [91], and single bolus injection of a fatty-acylated GIP receptor agonist into the third ventricle of mice is sufficient to decrease body weight [90]. In addition, the GIP/GLP-1 receptor co-agonist MAR709 decreases body weight in wild-type mice with superior potency over a pharmacokinetically matched selective GLP-1 receptor agonist, and this superiority disappears in mice with neuronal loss of GIP receptors [90]. While these data argue for a role of the brain GIP receptor in regulating food intake, they have all been collected in rodents. In human studies, exogenous GIP in pharmacological doses did not reduce food intake or visual analogue scale assessments of appetite, satiety and prospective food consumption [92, 93]; rather, GIP interfered with the otherwise robust reduction in energy intake caused by GLP-1 [92]. Thus, results from rodent studies contradict those from human clinical trials. Whether or not this is due to a veritable species difference needs to be clarified.

The ability of GIP receptor stimulation to contribute to improved glycaemic control (over and above that provided by the selective GLP-1 receptor agonist semaglutide) has been addressed in a dedicated mode-of-action study [94], but several controversies remain: (1) insulin sensitivity was improved more with tirzepatide than with the selective GLP-1 receptor agonist semaglutide, but weight loss was greater with tirzepatide, too; it remains to be determined whether, in human type 2 diabetes, GIP receptor agonism improves insulin sensitivity beyond the effects mediated by body weight reduction [95]; (2) while glucose-induced insulin secretion increased more with tirzepatide than with semaglutide (hyperglycaemic clamp experiments), insulin secretory responses to a test meal did not differ (while glucose excursions were lower with tirzepatide) [94]; (3) glucagon suppression after a test meal was more marked with tirzepatide than with semaglutide [94], while exogenous GIP added to GLP-1 prevented the (otherwise robust) suppression of glucagon in fasting patients who were hyperglycaemic at baseline [41]; (4) as GIP has no effects on gastric emptying [46], the deceleration of gastric emptying with tirzepatide [96] is most likely caused by GLP-1 receptor agonism.

Another open question regards the role of GIP receptor agonists vs GIP receptor antagonists in the reduction of body weight. Animal studies have suggested that both can be a successful approach to lowering body weight [97, 98]. Similar to GIP/GLP-1 receptor co-agonism, GLP-1 receptor agonism in combination with GIP receptor antagonism (either as co-therapy or using unimolecular formulations) improved glucose metabolism and decreased body weight in preclinical [99] and clinical studies [100]. Whether GIP receptor agonism and antagonism affect energy metabolism via similar or distinct mechanisms and target organs remains to be determined, as well as whether GIP receptor agonism may lead to receptor desensitisation, as previously hypothesised [101].

Several in vitro studies have further demonstrated that tirzepatide [102], MAR709 [103] and other compounds [104] that show GLP-1 as well as GIP receptor agonism may differ from selective GLP-1 receptor (mono-)agonists (including semaglutide and GLP-1 [7–36 amide]) by showing delayed internalisation and faster recycling of GLP-1 receptors. GLP-1 receptor agonists trigger signal transduction through G proteins leading to cAMP formation, but also induce the ß-arrestin pathway leading to GLP-1 receptor internalisation, which reduces the number of GLP-1 receptors on the cell surface and may impair prolonged GLP-1 receptor agonism. Biased agonism describes the fact that ligands of any G protein protein-coupled receptor may differ from the original ligand (e.g. GLP-1) in their intracellular signalling patterns, including recruitment of G proteins (G_as_ signalling to produce cAMP, or G_aq_ recruitment to initiate inositol trisphosphate signalling) or activation of ß-arrestin. However, how biased agonism at the GLP-1 receptor contributes to the metabolic efficacy of individual ligands has only been estimated by inference (assuming that more internalisation reduces the GLP-1 receptor number on the cell membrane and potentially leads to desensitisation). It may, however, become an important mechanism for improving the efficacy and durability of GLP-1 receptor agonists. In light of these studies, tirzepatide may, disregarding any contribution of GIP receptor agonism, be a particularly effective GLP-1 receptor agonist [105]. In any case, the clinical success of GLP-1-based drugs combined with agonism (e.g. tirzepatide [84]) or antagonism [100] at the GIP receptor, and of the even more advanced GIP/GLP-1/glucagon receptor triagonists [106], suggest that there is potential for further improvements in the effectiveness (for glycaemic control and weight reduction) of incretin-based medications for the treatment of type 2 diabetes, which might eventually challenge the role of bariatric surgery in inducing substantial weight reduction and type 2 diabetes remission.

## Conclusions

Incretin hormones (GIP and GLP-1) play an important role in the pathophysiology (reduced incretin effect) and progression (given the deterioration of postprandial glycaemic control as a result of the reduced incretin effect) of type 2 diabetes. However, GLP-1 has therapeutic potential, which has been successfully exploited by developing GLP-1 receptor agonists. While 35 years ago, GIP was judged to be devoid of any therapeutic potential, novel compounds such as tirzepatide, which also stimulates GIP receptors and has a remarkably improved effectiveness for controlling plasma glucose concentrations and reducing body weight (vs selective GLP-1 receptor agonists), have renewed interest and sparked studies into the novel roles of GIP in preventing ectopic fat deposition and improving insulin sensitivity and in reducing appetite and energy intake (to date shown only in animal models). In addition, dual and triple agonists that activate GLP-1 and other gastro-entero-pancreatic hormone receptors have obvious potential for synergistic effects and improved effectiveness. Thus, incretin hormones have proven to be useful parent compounds for therapeutic peptides, and the expectations regarding their use for the treatment of diabetes, obesity and associated cardiorenal complications are high.

## Supplementary Information

Below is the link to the electronic supplementary material.

Supplementary file1 (PDF 468 KB)

## Data Availability

Data extracted from included studies; data used for all analyses and any other materials used in the review can be accessed on reasonable request and at the discretion of the authors by contacting the corresponding author.

## Abbreviations

apoB48: Apolipoprotein B-48
DPP-4: Dipeptidyl peptidase-4
GIP: Glucose-dependent insulinotropic polypeptide
GLP-1: Glucagon-like peptide-1
IFG: Impaired fasting glucose
IGT: Impaired glucose tolerance
NGT: Normal glucose tolerance
