# Supplementary material for: Incretin hormones and type 2 diabetes
Source: Diabetologia. 2023 Jul 11;66(10):1780–95. doi: 10.1007/s00125-023-05956-x (PMC10474001; doi:10.1007/s00125-023-05956-x)
Supplement: Supplementary file 1 — Supplementary file1 (PDF 468 KB) [file 125_2023_5956_MOESM1_ESM.pdf]

ELECTRONIC SUPPLEMENTARY MATERIAL

Incretin hormones and type 2 diabetes

Michael A. Nauck[1], Timo D. Müller[2,3]

[1]Diabetes, Endocrinology, Metabolism Section, Medical Department I, Katholisches Klinikum Bochum, St. Josef Hospital, Ruhr-University Bochum, Bochum, Germany; [2]Institute for Diabetes and Obesity, Helmholtz München, Neuherberg, Germany [3]German Center for Diabetes Research (DZD), Neuherberg, Germany

ESM Table 1: Subject characteristics of participants with type 2 diabetes or non-diabetic control subjects in meta-analyses comparing glucose-dependent insulinotropic polypeptide (GIP) and glucagon-like-1 (GLP-1) plasma responses (total area under the curve) after nutrient intake (oral glucose, liquid or solid meals) reported by Calanna et al. (see Figure 2)

		GIP_{total}		$GLP\text{-}1_{total}$	
		Type 2 diabetes	Non-diabetic controls	Type 2 diabetes	Non-diabetic controls
Number of studies	n	22		22	
Number of patients	n	363	325	275	279
Proportion female	[%]	51	53	46	47
Age	[years]	54 (18-71)	50 (19-71)	55 (18-76)	49 (19-71)
HbA_{1c}	[%]	7.4 (6.1-9.2)	5.4 (3.6-6.5)	7.2 (5.2-9.2)	5.5 (4.0-6.4)
	[mmol/mol]	57 (42-75)	31 (9-42)	55 (33-77)	37 (20-46)
Fasting plasma glucose	[mmol/l]	9.1 (5.3-12.0)	5.3 (4.6-6.0)	8.0 (5.0-12.0)	5.0 (4.0-6.4)
Body-Mass-Index	[kg/m^2]	34 (24-52)	30 (22-46)	33 (23-46)	30 (22-46)
Fasting GIP	[pmol/l]	18.8 (5.7-45.0)	17.6 (4.5-49.0)	n.r.	n.r.
Fasting $GLP\text{-}1_{total}$	[pmol/l]	n.r.	n.r.	12.5 (5.8-23.4)	12.6 (4.9-31.0)

Means, ranges; n.r.: Not reported

ESM Table 2: Studies comparing insulinotropic effects of glucose-dependent insulinotropic polypeptide (GIP) and glucagon-like peptide-1 (GLP-1) in patients with type 2 diabetes and in healthy control subjects (see Figure 3): Patient characteristics and methodological details (hyperglycaemic clamp plasma glucose concentrations and duration; GIP and GLP-1 infusion rates and steady state plasma concentrations)

Study		Sex	Age	Diabetes duration	Glycated haemoglobin (HbA_{1c})	Body-Mass-Index	Diabetes treatment	GIP infusion rates	GIP_{total} plasma concentrations (steady state)	GLP-1 infusion rates	GLP-1_{total} plasma concentrations (steady state)	Plasma glucose (clamp)	Duration of GIP/GLP-1 infusion
First author/ year	Group	Female/ male	[years]	[years]	[%]	kg/m^2	Generic drug (class) names	$pmol \cdot kg^{-1} \cdot min^{-1}$	pmol/l	$pmol \cdot kg^{-1} min^{-1}$	pmol/l	mmol/l	h
Nauck et al. 1993	Type 2 diabetes patients	4/5	59 ± 10	8 ± 9	6.3 ± 0.6	28.6 ± 3.1	Diet, sulfonylureas[a]	0.8; 2.4	~ 450; ~ 1100	0.4; 1.2	~ 100; ~ 250	8.0	1
	Healthy controls[b]	5/4	59 ± 11	-	5.5 ± 0.2	26.8 ± 4.1	-	0.8; 2.4	~ 500; ~ 1200	0.4; 1.2	~ 100; ~ 220	8.0	1
Vilsbøll et al. 2002	Type 2 diabetes patients	1/7	55	6	7.4	29.5	Diet, metformin, sulfonylureas[a]	4; 16	~ 350; ~ 2200	1	~ 125	15.0	4 (2)[c]
	Healthy controls[b]	1/5	54	-	5.6	29.6	-	4	~ 350	1	n.r. (n=1)	15.0	2
Højberg et al. 2009	Type 2 diabetes patients	n.r.	52 ± 9	5 ± 4	8.6 ± 1.3	31.1 ± 3.9	Metformin, sulfonylureas[a]	1.5	~ 130	0.5	~ 45	15.0	2
	Healthy controls[b]	n.r.	51 ± 9	-	5.3 ± 0.2	30.8 ± 3.9	-	1.5	~ 145	0.5	~ 45	15.0	2

Means ± SD; [a]: discontinued before the study; [b]: Normal oral glucose tolerance test; [c]: Results concerning insulin secretory responses between type 2 diabetes patients and healthy control subjects were compared for a 2 h duration; n.r.: Not reported

ESM Table 3. Patient characteristics of subjects with type 2 diabetes in whom the incretin effect was quantified (data presented in Figure 6)

	Publication (First author, year, reference)	Sex (female/ male)	Age [years]	Known duration of diabetes [years]	Body-mass-index [kg/m^2]	Fasting plasma glucose [mmol/l]	120 min plasma glucose after oral glucose [mmol/l]	HbA_{1c} [%]	Glucose-lowering medications
1	Nauck et al. 1986	7/7	61 ± 11	7 ± 7	25.2 ± 3.1	8.3 ± 2.4	15.1 ± 3.3	n.r.	S (n = 5)
2	Knop et al. 2007	2/6	62 (51-75)	n.r.	24 (21-26)	8.1 (6.2-10.4)	~ 12.3 [a]	6.8 (6.2-8.7)	S (n = 4), M (n = 1)[b]
3	Bagger et al. 2011	5/3	57 (40-75)	1 (0.5-3)	29 (25-34)	7.7 (7.0-8.9)	~ 15.5 [a]	7.0 (6.2-8.4)	none
4	Vardarli et al. 2011 [c]	3/18	59 ± 9	6 ± 3	28.6 ± 2.6	9.3 ± 1.1	~ 16.0 [a]	7.3 ± 0.5	M (n= 21)
5	Vardarli et al. 2014 [c]	4/16	59 ± 7	5 ± 3	30.6 ± 3.0	~ 8.1 [a]	~ 15.5 [a]	7.0 ± 0.6	M (n = 15), S (n = 1)
6	Laferrere et al. 2007 [c]	8/0	45 ± 10	2 ± 1	43.4 ± 6.8	8.1 ± 1.8	~ 12.2 [a]	6.9 ± 0.7	M or S [e]

Means ± standard deviation or median and interquartile range; n.r.: not reported; S: Sulfonylureas; M: metformin; [a]: Read from figures; [b]: Combination of S and M in 1 patient; [c]: At screening (before DPP-4 inhibitor treatment) [d]: Before gastric bypass surgery; [e]: Discontinued 3 days before experiments.

References quoted in the online supplement

[1] Faerch K, Torekov SS, Vistisen D, et al. (2015) GLP-1 response to oral glucose is reduced in prediabetes, screen-detected type 2 diabetes, and obesity and influenced by sex: The ADDITION-PRO Study. Diabetes 64: 2513-2525

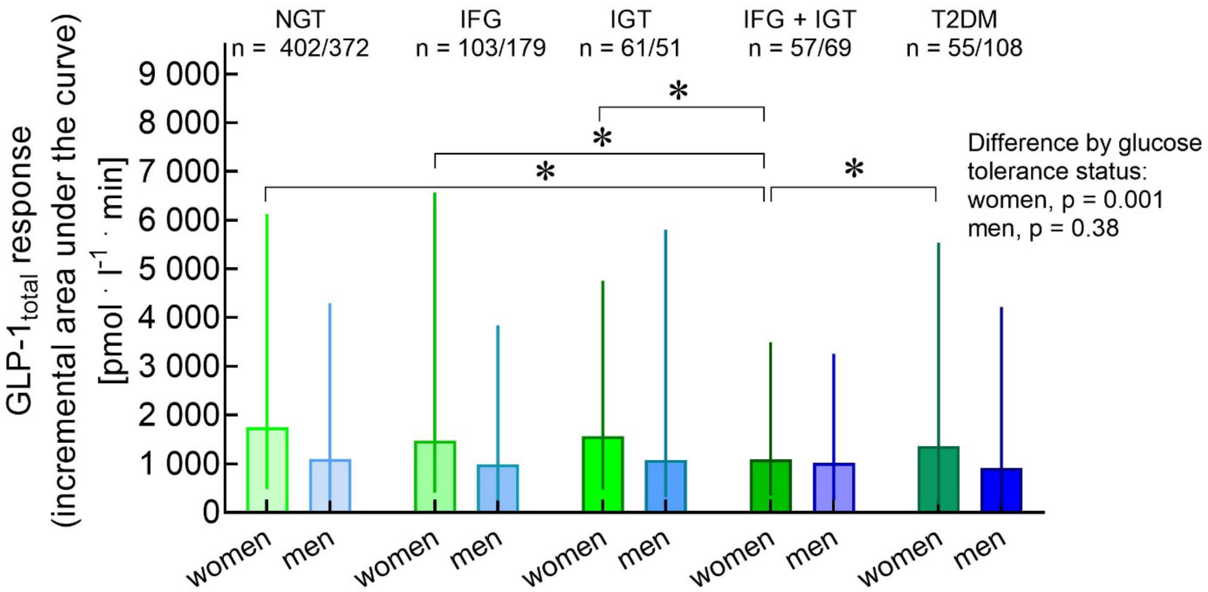

ESM Figure 1. Secretion of GLP-1 in patients with type 2 diabetes relative to subjects with no impairment in glucose tolerance or various categories of prediabetes (IFG; impaired fasting glucose; IGT: impaired glucose tolerance; IFG/IGT: Combination of impaired fasting glucose. And impaired glucose tolerance. Significant differences in incremental GLP-1_{total} plasma responses following an oral glucose load across categories of glucose tolerance (including screen-detected type 2 diabetes) in women and men (Faerch et al. 2015 [1]). P-values are presented for the overall comparison between all groups by ANOVA (without any adjustment). Asterisks indicate significant differences in iAUC for total GLP-1 ($p < 0.05$) between columns connected by brackets. Only in women, significant differences were found for selected comparisons. Inter-individual variability was high, as indicated by wide 95 % confidence intervals, in both women and men. Total AUCs (see Figure 2 B and C) did not display any significant differences by glucose tolerance status.
